# Ultrastructure of *Meelsvirus*: A nuclear virus of arrow worms (phylum Chaetognatha) producing giant “tailed” virions

**DOI:** 10.1371/journal.pone.0203282

**Published:** 2018-09-19

**Authors:** George L. Shinn, Brianna L. Bullard

**Affiliations:** Biology Department, Truman State University, Kirksville, Missouri, United States of America; Centro Nacional de Biotecnologia (CNB-CSIC), SPAIN

## Abstract

Most known giant viruses, i.e., viruses producing giant virions, parasitize amoebae and other unicellular eukaryotes. Although they vary in the level of dependence on host nuclear functions, their virions self-assemble in the host cell’s cytoplasm. Here we report the discovery of a new prototype of giant virus infecting epidermal cells of the marine arrow worm *Adhesisagitta hispida*. Its 1.25 μm-long virions self-assemble and accumulate in the host cell’s nucleus. Conventional transmission electron microscopy reveals that the virions have a unique bipartite structure. An ovoid nucleocapsid, situated in a broad “head” end of the virion is surrounded by a thin envelope. The latter extends away from the head to form a voluminous conical “tail” filled with electron-dense extracapsidular material. The 31nm-thick capsid wall has a distinctive substructure resulting from a patterned arrangement of subunits; it bears no ultrastructural resemblance to the virion walls of other known giant viruses. The envelope self-assembles coincident with the capsid and remotely from all host membranes. We postulate that transmission to new hosts occurs by rupture of protruding virion-filled nuclei when infected arrow worms mate. Future genomic work is needed to determine the phylogenetic position of this new virus, which we have provisionally named *Meelsvirus*.

## Introduction

“Giant viruses” are distinguished by the large physical size and/or large genome content of their virions (infective virus particles) [1, 2]. The largest known virions, produced by *Mimivirus*, *Pandoravirus*, *Pithovirus*, *Mollivirus*, and *Tupanvirus*, exceed 0.3 μm and are clearly visible by light microscopy [3–7]. Viruses producing giant virions do not constitute a monophyletic group [1, 2, 6, 8–12]. Based on morphological and molecular differences between them, Abergel, Legendre & Claverie [12] concluded that *Mimivirus*, *Pandoravirus*, *Pithovirus*, and *Mollivirus* are no more closely related to each other than any of them is to the last common ancestor of cellular organisms. They may have originated separately from different ancestral protocells [12]. Only a few dozen genera of giant and near-giant viruses have been reported to date, but it is predicted that a huge diversity of giant viruses exists and that they infect a broad array of host types in all kinds of environments, including the oceans [1, 2].

Broadening our knowledge of giant viruses depends, in part, on the chance discovery of new “prototypes” of giant viruses [1]. When a new prototype is discovered, sampling methods can be developed that target similar giant viruses. For example, once *Mimivirus* was discovered to be a virus (rather than bacterium) and that its *Acanthamoeba* hosts become infected by phagocytosing virions [3], amoebae were used as “traps” thereby enabling discovery of other giant amoeba-infecting viruses, including *Pandoravirus*, *Pithovirus*, *Mollivirus*, and *Tupanvirus*, as well as numerous additional strains of *Mimivirus* [4–8]. Likewise, once a new prototype of giant virus is discovered, unique genomic sequences can be identified for use as molecular probes in metagenomic analyses of environmental samples [13–16]. Because of the specificity of sampling methods employed to date, most known giant viruses are parasites of amoebae and other unicellular eukaryotes [2, 8].

This paper reports the serendipitous discovery of a new prototype of giant virus infecting epidermal cells of the marine arrow worm *Adhesisagitta hispida*. We have used transmission electron microscopy to describe the unique morphology of its virions, self-assembly of virions within the host cell nucleus, and major structural transformations occurring in infected host cells. Distinguishing features include an ovoid capsid with a distinctively patterned non-layered substructure, simultaneous self-assembly of an envelope around the forming capsid, and elaboration of the envelope to form a “filled” conical tail. Because the distinctive shape and large physical size of the virions bring to mind traditional Persian meels, we propose the genus name *Meelsvirus* for this new virus. We postulate that viral transmission occurs when hosts mate. All of our specimens were preserved in glutaraldehyde and embedded in Epon resin for sectioning; we have no genetic information about the virus.

The arrow worms hosts of *Meelsvirus* belong to the phylum Chaetognatha. Arrow worms are of broad biological interest because they are ecologically important predators of marine zooplankton [17–21] and because their evolutionary relationships to other animal phyla remain unresolved [18, 22–24]. To our knowledge, this is the first virus to be described from arrow worms [25].

## Methods and materials

### Collection of hosts

The host of *Meelsvirus* is an invertebrate worm species that is neither endangered nor protected in any way and no written permission was required for its collection or for subsequent handling in the laboratory. At the time the live hosts were collected, kept in the laboratory, and subsequently preserved for electron microscopy (e.g., 1985, 1988), no animal use review process existed at Harbor Branch Oceanographic Institution and no approval was required for the collection or preservation of the study organisms.

The virus was discovered by chance during the senior author’s ultrastructural studies of the chaetognath, *Adhesisagitta hispida*. Of the several dozen, apparently healthy, adult chaetognaths that were examined by transmission electron microscopy, two were found to be infected. One host was collected during April 1986, the other during June 1988.

The chaetognaths were obtained from the Indian River Lagoon, Florida (27° 14’N; 80° 90’ W; 0–5 m depth). A 300 μm mesh, ¼ meter diameter plankton net with a closed cod end was dropped into the tidal current from a fishing pier. Tows were kept short (3–5 minutes at a time). Chaetognaths were separated from the rest of the captured plankton immediately after each tow. In order to eliminate specimens that were damaged during collection, the chaetognaths were kept alive in the laboratory for 1–2 days in 5” covered specimen bowls at 15–17°C. They were fed copepods that had been collected along with the chaetognaths. Because the goal at that time was to obtain healthy specimens for study of chaetognath tissues, any virus-infected animals that looked unhealthy would have been discarded along with other moribund specimens.

### Fixation, embedding, and staining

Chaetognaths were killed instantaneously by immersion in Karnovsky’s paraformaldehyde-glutaraldehyde fixative in 0.2 M Sorenson’s phosphate buffer (room temperature, 1–2 h) [26]. Immediately after immersion, a sharp razor blade was used to cut each specimen transversely into several short pieces. This allowed rapid penetration of the fixative and greatly improved the quality of fixation. Specimens were post-fixed in 2% OsO_4_ in 0.2 M phosphate buffer (room temp, 1 h), then dehydrated in an ethanol series, transferred through three changes of propylene oxide, and embedded in Epon 812. Thin sections were stained in a saturated solution of uranyl acetate (30 minutes) followed by Reynolds’ lead citrate stain (5 minutes) [27].

### Electron microscopy

Thin sections were studied with a JEOL JEM-100SX transmission electron microscope. Images were captured with a bottom-mounted, 1 megapixel AMT digital camera. Image J was used to take measurements from digital images captured at 10,000X. Within image J, the scale was set using digital images of a calibration grid that were also captured at 10,000X.

## Results

### Infected host cells

**Fig 1:**
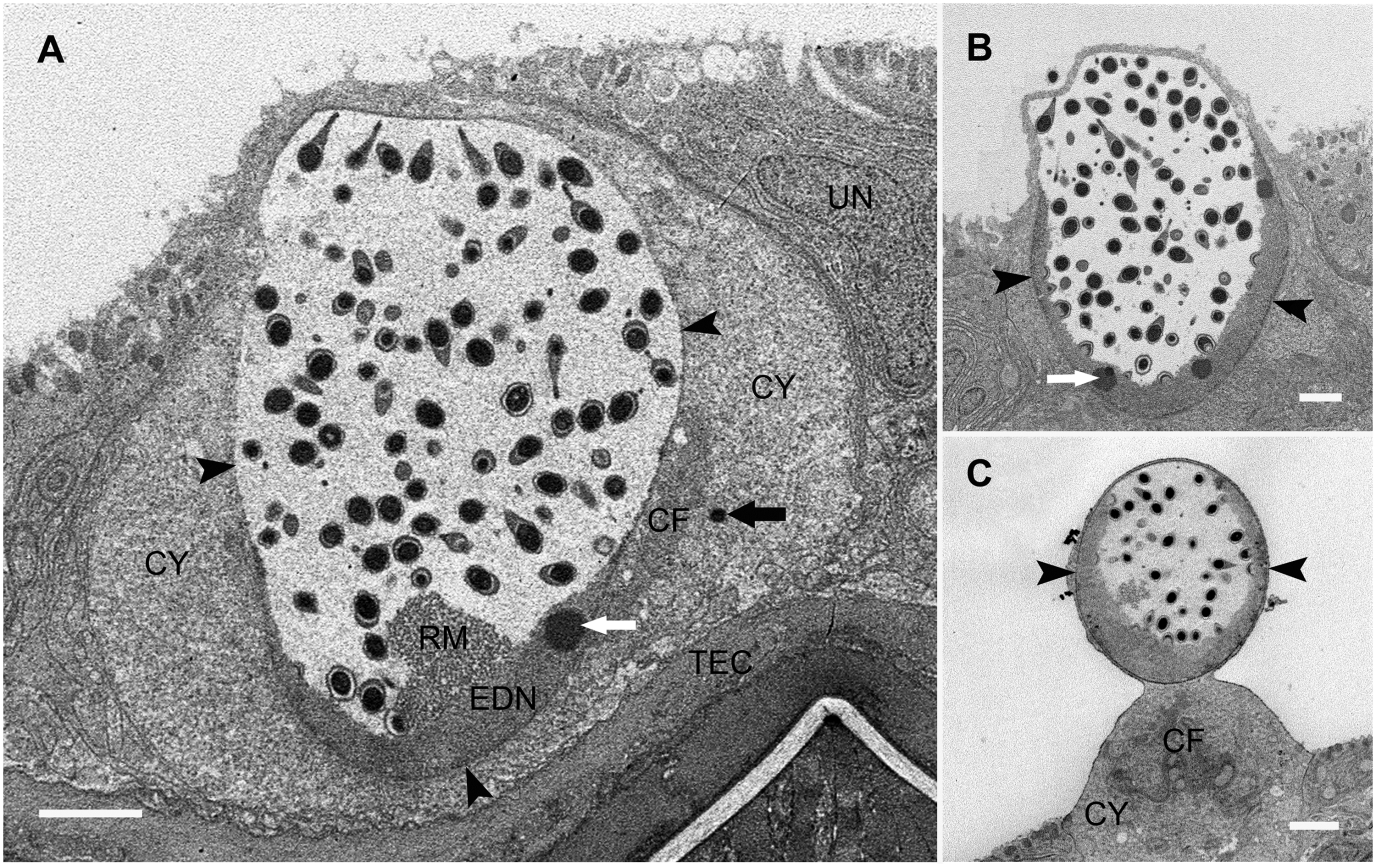
Hypertrophied virus-infected nuclei in surface epidermal cells of *Adhesisagitta hispida*. **A**. Virus-infected nucleus located centrally within cell. **B**. Partially protruding virus-infected nucleus. **C**. Virus-infected nucleus protrudes above the rest of its epidermal cell. Arrowheads indicate locations of nuclear envelope; CF, aggregates of cytoplasmic filaments; CY cytoplasm; EDN, electron dense nucleoplasm; RM, reticulate mass; TEC, tonofilament-rich epidermal cells; UN, nucleus of uninfected surface gland cell; black arrow indicates full naked capsid in cytoplasm; white arrow indicates electron dense body. Scale bars = 2 μm.

*Meelsvirus* infects surface gland cells of the host’s stratified squamous epidermis (Fig 1). Infected cells are interspersed with normal-looking surface gland cells and underlain by normal-looking tonofilament-rich epidermal cells. These cells are conspicuous even at low magnifications of the TEM because their nucleus is hypertrophied and spheroidal rather than flattened (Fig 1). The position of the nucleus varies among infected cells. In six cells, it is situated centrally; in the other six, it bulges apically. In one extreme case, the nucleus and a very thin surrounding rim of cytoplasm is almost completely separated from the main body of the cell (Fig 1C). For both hosts, infected cells are located in a small dorso-lateral area in the posterior half of the body. In 33 thin sections through the infected areas, we found 12 individually recognizable infected cells. Other tissues at the sectioned level of the body, such as the body wall muscles and spermatogenic cells, appear normal and show no signs of the virus.

**Fig 2:**
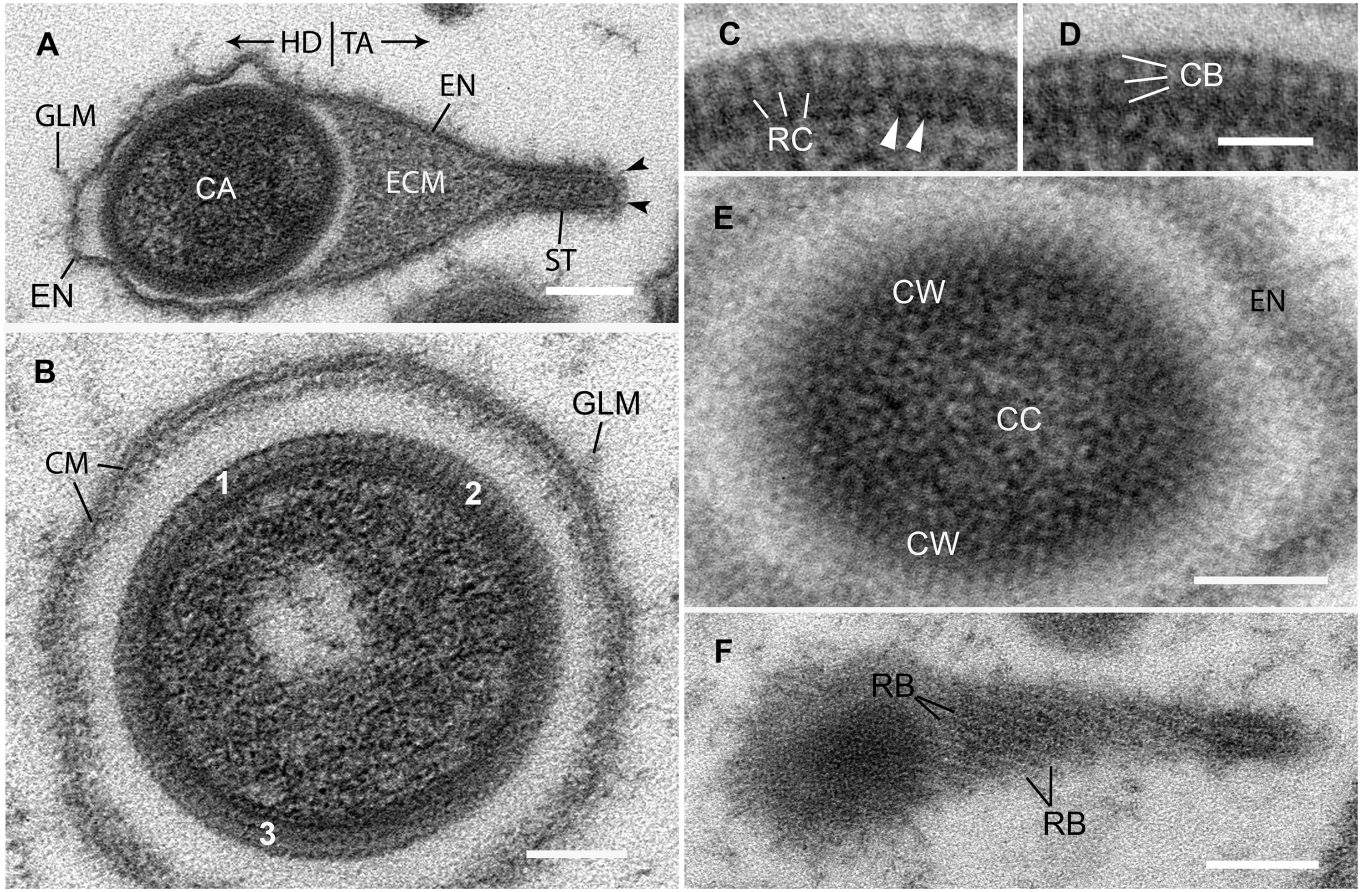
*Meelsvirus*, structure of mature virions. **A**. Longitudinal section through entire virion, showing head (HD) and tail (TA). The envelope (EN) surrounds the capsid (CA) and extends over the surface of the tail. ECM, extracapsidular material in tail; GLM, glycocalyx-like material; ST, stem of tail; arrowheads indicate tail spikes. Scale bar = 0.2 μm. **B**. Cross section through head. The capsid wall varies in appearance depending on the exact plane of section through it. Area 1 is sectioned perpendicular to the surface; areas 2 and 3 have been somewhat obliquely cut. CM, central membrane of envelope; GLM, glycocalyx-like material. Scale bar = 0.1 μm. **C-D**. Details of capsid wall. RC, radial columns; CB, cross bridges; arrowheads indicate punctae. Scale = 0.05 μm. **E**. Tangential section through capsid wall showing patterned substructure. CC, capsid contents; CW, capsid wall; EN, envelope. Scale bar = 0.1 μm. **F**: Tangential longitudinal section of virion, showing ribs (RB). Scale bar = 0.2 μm.

**Fig 3:**
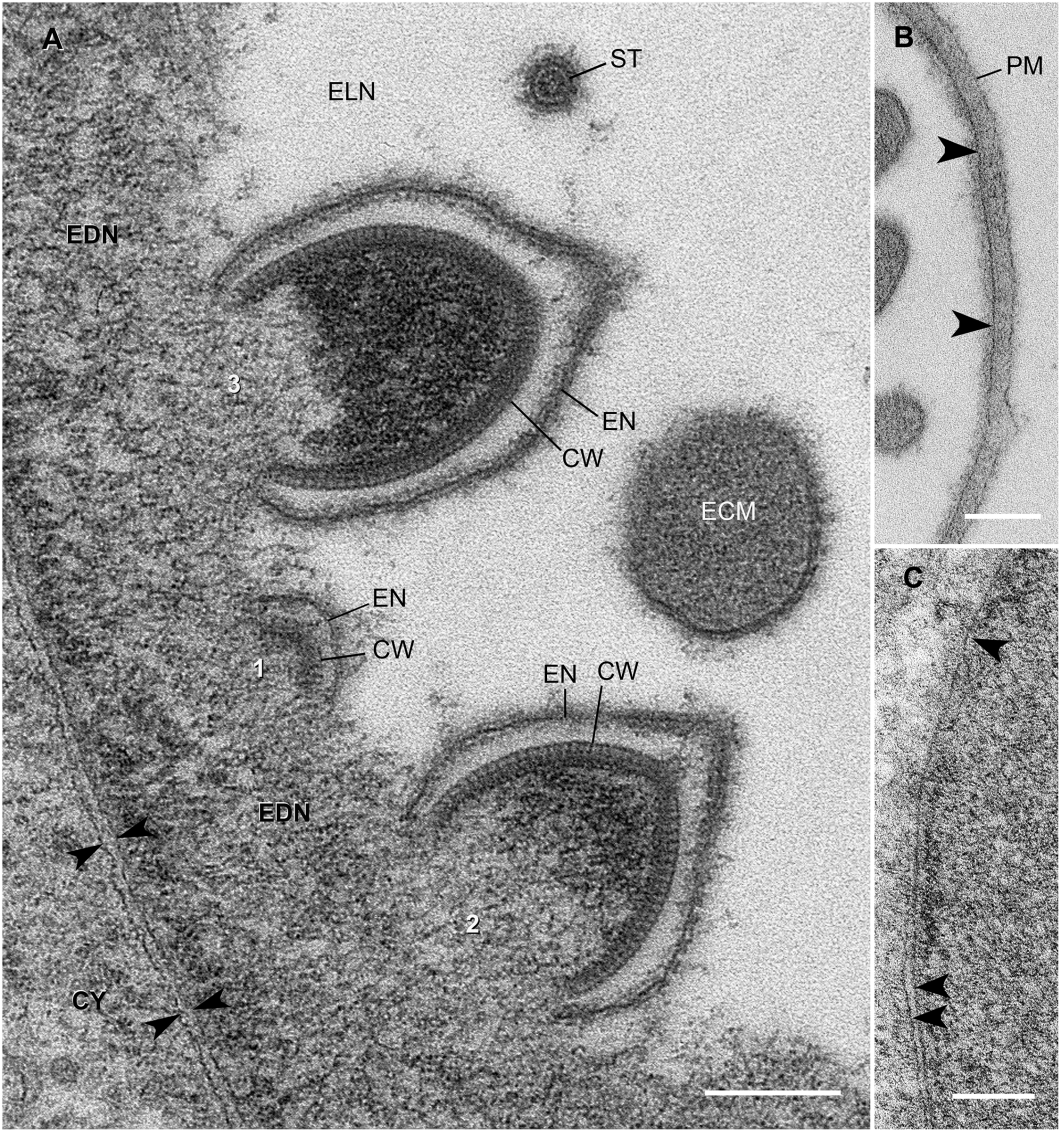
Details of infected nuclei and forming virions. **A**. Basal edge of virus-infected nucleus. Numbers (1, 2, 3) show successive stages of virion assembly on the surface of the electron dense nucleoplasm (EDN). Note that the envelope and capsid wall self-assemble simultaneously. CW, capsid wall; CY, cytoplasm; ECM, extracapsidular material in a cross-sectioned tail; ELN, electron lucent nucleoplasm; EN, envelope; ST, stem of tail. **B**. Apical side of nucleus, showing single membrane of nuclear envelope (arrowheads) adjacent to plasma membrane (PM). **C**. Nuclear envelope in area where outer membrane becomes discontinuous. Arrowheads indicate membranes of the nuclear envelope. Scale bars = 0.2 μm.

**Fig 4:**
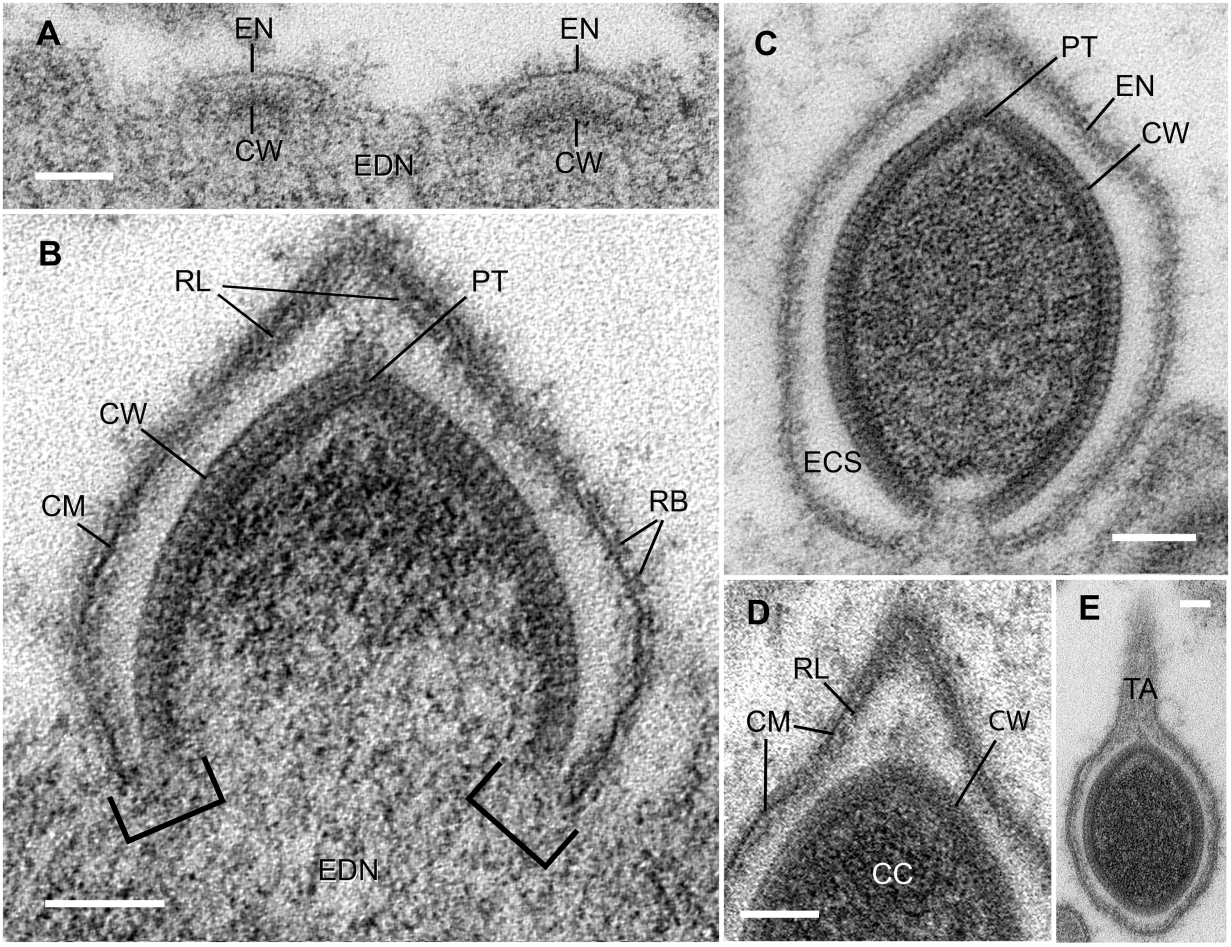
Successive stages of virion self-assembly. **A**. During the earliest stages of virion formation, the envelope (EN) and capsid wall (CW) self-assemble on the inner surface of the electron dense nucleoplasm (EDN). **B**. Mid-stage of virion formation. Brackets indicate site of self-assembly of capsid wall and envelope. CM, central membrane of envelope; CW, capsid wall; EDN, electron dense nucleoplasm; PT, possible portal-like specialization of capsid wall; RL, reinforcing layer of forming tail stem; RB, ribs. **C**. Latest stage of virion formation that is associated with the inner surface of the electron dense nucleoplasm. The capsid wall (CW) and envelope (EN) are nearly closed. The envelope is separated from the capsid wall by a lucent extracapsidular space (ECS). PT, possible portal-like specialization of capsid wall. **D**. Detail of forming tail-end of virion. CC, capsid contents; CM, central membrane of envelope; CW, capsid wall; RL, reinforcing layer of forming tail stem. **E**. Longitudinal section of nearly-formed virion, showing envelope extended as a narrow tail (TA), but tail apparently not yet filled with extracapsidular material. Scale bars = 0.1 μm.

All infected cells in both host specimens appear to be at a similar stage of viral “attack.” The nuclei contain mature virions suspended in an abundant electron-lucent nucleoplasm (Figs 1 and 2). Single thin sections through individual nuclei reveal as few as 28 and as many as 95 mature virions. The nuclei also contain forming virions at various stages of self-assembly (Figs 3 and 4). The latter are arranged along the inner surface of a thick layer of peripheral electron-dense nucleoplasm (EDN). Except apically, the EDN borders the nuclear envelope and includes several conspicuous spherical electron dense bodies and, commonly, an adjacent irregularly shaped reticulate mass (Fig 1A).

Where it is bordered by electron dense nucleoplasm, the nuclear envelope has the structure typical of eukaryotic cells: inner and outer membranes are separated by a perinuclear cisternal space and the two nuclear membranes are continuous at nuclear pores (Fig 3A). Along the apical side of the nucleus, one or both membranes of the nuclear envelope are discontinuous or absent (Fig 3B and 3C). In these areas, however, the nuclear contents remain segregated from the cytoplasm.

Free ribosomes, mitochondria, and small sectional profiles of endoplasmic reticulum dominate the cytoplasm of infected cells. Most infected cells contain a few apical secretory granules resembling those in uninfected surface cells. Cells with an apically bulging nucleus commonly have conspicuous, irregularly shaped aggregates of cytoskeletal filaments in the more basal cytoplasm (Fig 1A and 1C).

### Structure of mature virions

Virions of *Meelsvirus* have a distinctive bipartite form: a head containing the ovoid nucleocapsid joins a conical tail, which terminates in a narrow straight-sided stem (Fig 2A–2F). Mature virions average 1.25 μm long (range: 1.03–1.37 μm). The nucleocapsid, a prolate spheroid, is 0.53 μm long (range: 0.48–.56 μm) and 0.40 μm wide (range 0.36–0.46 μm).

The capsid wall is 31 nm thick (range: 29–33 nm). It has a complexly patterned substructure due to the regular arrangement of component parts (Fig 2B–2E). The following details can be resolved in favorable parts of sections cut perpendicular to the capsid surface (Fig 2C and 2D). Radially oriented electron-dense columns span the entire thickness of the capsid wall. These are spaced approximately 13 nm apart and are thinner than the less electron-dense spaces between them. At the outer edge, near the middle, and at the inner edge of the capsid wall, electron-dense elaborations form cross-connections between adjacent columns. The inner-edge cross-connections are especially electron dense and appear to form a “floor” from which the radial columns arise. Also, electron dense punctae project centrally from the inner edge of the capsid wall. Subtle variations in the plane of section strongly affect the appearance of the wall, especially when viewed at low magnifications (Fig 2B). In some views, the cross-connections are inconspicuous. In others, the cross-connections are more conspicuous than the radial columns, giving the mistaken impression that the capsid wall is composed of three electron dense laminae separated by two electron lucent laminae (Fig 2B). Tangential sections through the capsid wall show a variety of patterns that change over very short distances (Fig 2E). Electron dense spots presumably represent cross sections through individual radial columns. The regular arrangement of these spots (i.e., radial columns) is especially apparent at the periphery of tangentially sectioned capsids, where they form evenly spaced, slightly curving rows. In other places, electron dense components of the wall form short rods variously arranged as zig-zags, honeycomb-like arrays, and crosses. We interpret these rods to be the cross-connections between adjacent radial columns.

A portal-like specialization may exist in the end of the capsid bordering the tail. The patterned structure of the capsid wall is routinely obscure at this site. Also, amorphous electron dense material projects from this area into the lucent space separating the capsid from the contents in the tail (Fig 4B and 4C).

Electron-dense, fibro-granular material evenly fills most capsids. These contents adhere very closely to the inside of the capsid wall. Most capsids also contain one or two spheroidal areas where the contents are less-densely packed (Fig 2B). In occasional virions, some of the contents are arranged as regularly spaced, high-density masses.

A thin envelope bounds the entire virus. On the head, it is commonly wrinkled and separated from the capsid by a lucent gap (Fig 2A). The long axis of the nucleocapsid typically parallels the long axis of the virion, but it is tilted somewhat within the envelope of some virions. The envelope always adheres closely to the contents of the tail and stem.

The envelope has an electron dense “central membrane”, decorated on both sides by additional adhering material (Figs 2B, 3A and 4B). The central membrane is similar in thickness to the unit membranes of the nuclear envelope and, therefore, may be a unit membrane. At high magnification (30,000x), we were not able to verify a trilaminar construction, but neither could we verify the trilaminar structure of the nuclear membranes or other membranes in the host cells.

Electron dense material, arranged as parallel "ribs", adheres to the outside of the central membrane. These are tilted at a slight angle to the long axis of the virion (Fig 2F). Where cross-sectioned, the ribs protrude block-like above the central membrane (Fig 4B). Where cut longitudinally, they appear to form a discrete layer on the outside of the central membrane. Where the envelope is lifted off of the capsid, electron dense material adhering to the inside of the central membrane usually looks fuzzy. On both head and tail, the envelope is further decorated externally by scattered elongate glycocalyx-like macromolecules that extend out into the nucleoplasm (Fig 2A and 2B).

The tail is a conical elaboration of the envelope filled with extracapsidular material (Figs 2A and 3A). The latter is evenly granular and, typically, less electron dense than the contents of the capsid. The wide end of the tail conforms to the width of the capsid. At the narrow end of the tail, the stem is distinguished by its narrow uniform width and presence of a central cylindrical core. The stem measures 252 nm long (range: 192–292 nm) x 75 nm wide (range: 64–80 nm). Favorable longitudinal sections show straight spikes radiating from the end of the tail.

### Assembly

The envelope and capsid wall self-assemble simultaneously (Figs 3 and 4). They arise from the inner surface of the peripheral electron-dense nucleoplasm (EDN; Fig 3). The free-edges of forming capsids and envelopes are embedded in the surface of the EDN, giving the impression that they grow by accretion of materials from the adjacent EDN. The envelope and capsid of the earliest virions border each other closely and their growing edges remain close through all stages of formation. As a virus enlarges, the envelope increases in surface area faster than the capsid, resulting in the formation of a large, empty-looking extracapsidular space between them (Fig 4C). Although forming virions have no obvious tail, evidence suggests that the future tail end of the virus forms first. Favorable sectional profiles show the capsid’s portal-like specialization at this end. Also, the adjacent envelope is conical due to presence of an inner reinforcing layer, suggestive of a forming tail stem (Fig 4D).

As the enlarging capsid rises above the EDN, some of the EDN is drawn into the capsid’s cavity. Once the capsid is half formed, the contents at the closed end become much more electron dense, suggesting condensation (packaging) of the contents (Figs 3A and 4B).

Because we never found virions with elongated tails associated with the EDN, we surmise that virions separate from the EDN before the tail lengthens and fills and before the stem matures. Some virions free in the lucent nucleoplasm had unusually thin tails (Fig 4E); others had unusually short tails. Either or both of these may represent intermediate stages of tail formation (tail filling). The source of the tail’s extracapsidular material remains uncertain.

Capsids sometimes self-assemble without an envelope and, consequently, never form a tail. Thus, occasional “naked” nucleocapsids are found suspended in the electron lucent nucleoplasm.

### Cytoplasmic virions

We surveyed the cytoplasm of both normal and infected cells for the possible presence of early stages of infection. Of the dozens of normal looking surface gland cells that we examined, only one contains evidence of very recent viral infection. The cytoplasm contains a single full, naked capsid on the apical side of the flattened normal-looking nucleus. We found no remnants of the envelope or tail. Examination of serial sections revealed that virtually all cells with an obviously infected nucleus have multiple naked capsids in the cytoplasm. Most of these capsids appear empty, but a few remain filled. Because the wall of empty capsids remains intact, we assume that the genetic material exits through a pore or portal in the capsid wall, but none of our sections shows an opening. One cell with an obviously infected nucleus has several entire virions in its cytoplasm. We suspect that these reached the cytoplasm through a conspicuous nearby rupture in the nuclear envelope.

## Discussion

We recognize *Meelsvirus* as a virus because the virions form by self-assembly [28]. Based on their unique morphology, we believe *Meelsvirus* to be a previously undiscovered virus and, possibly, the prototype of a new family of viruses. Its virions have the following distinctive combination of morphological attributes:

1. Large overall size.
2. Ovoid capsids, having a thick, non-layered, distinctively patterned wall.
3. Elaboration of an envelope to form a stemmed conical tail extending from one end of nucleocapsid.
4. Self-assembly of the envelope coincident with self-assembly of the capsid.

Our glutaraldehyde-fixed Epon-embedded specimens cannot be used for genomic analysis. Consequently, all of the most basic molecular questions about *Meelsvirus* remain unresolved. In the following morphological analysis, we compare *Meelsvirus* to the “true giants” as defined by the large physical size of their virions (i.e., easily visible by light microscopy, smallest dimension >0.3um) [12]. These are *Mimivirus*, *Pandoravirus*, *Pithovirus*, *Mollivirus*, and *Tupanvirus*.

### Large overall size

With a total length of 1.25 μm, *Meelsvirus* is among the largest known viruses [12, 29]. Even excluding the tail, its 0.5 μm long nucleocapsid is similar in physical size to those of other recently described giant viruses. The nucleocapsids of *Mimivirus*, *Mollivirus*, *and Tupanvirus* are about 0.5 μm in diameter [6, 12]; *Pandoravirus* is 0.7–1.5 μm long [4, 12]; and *Pithovirus* is 1–1.2 μm long. Among viruses, very large physical size sometimes coincides with very large genomic size [30]. Thus, *Meelsvirus* may have a very large genome including, potentially, a large gene repertoire.

### Ovoid capsids having a thick, non-layered, distinctively patterned wall

For *Meelsvirus* and other viruses with unusually large virions, large capsid volume coincides with presence of a thick, elaborate wall (also called a shell, tegument, or integument) [12]. Large internal volume presumably necessitates an especially thick wall in order to achieve structural integrity. Conventional TEM reveals that wall thickness is achieved in different ways in different lineages of these viruses, providing one clue that giant-sized virions have evolved independently in different virus lineages [5].

Compared to other viruses with unusually large virions, the patterned sub-structure of *Meelsvirus* capsids is strikingly visible. Our electron micrographs reveal that new capsids of *Meelsvirus* form by self-assembly of subunits located at the surface of the electron dense nucleoplasm. Taking capsomers to be the largest repeating subunits of a capsid, we conclude that the capsomers of *Meelsvirus* are unusually large. They appear to span the entire 31 nm thickness of the capsid, with no closely adhering additional layers on either side. We assume that the capsid of *Meelsvirus* is composed of proteins and that, as in many other viruses with very large virions, each capsomer is an assemblage of multiple close-fitting protein molecules [31–33]. Cryoelectron microscopy paired with three dimensional image reconstruction will be needed to further elucidate the molecular organization of the *Meelsvirus* capsid and biochemical analyses are needed to determine whether or not the putative capsomer proteins are homologous to those of any known viruses.

*Mimivirus* and numerous other large DNA viruses have virions with a capsomer substructure but, in contrast to *Meelsvirus*, their capsomers are not individually visible when viewed by conventional TEM [9, 32, 34, 35]. In *Mimivirus*, for example, the capsomers, which are trimers of major capsid protein, form a simple homogeneous, electron dense layer [35, 36]. At 31 nm thick, the capsid wall of *Meelsvirus* is about four times thicker than that of *Mimivirus* [32]; thus, capsomers of *Meelsvirus* are much larger than those of *Mimivirus*. Capsids of *Meelsvirus* differ from those of *Mimivirus* and related viruses in other ways—they are oval rather than pseudo-icosahedral in shape and they lack the long radiating surface fibers [36].

*Meelsvirus* capsids resemble virions of *Pandoravirus* and *Pithovirus* in having an ovoid shape, but the details of wall structure are conspicuously different. Likewise, the spheroid capsids of *Mollivirus* differ conspicuously in wall structure. In *Pandoravirus and Mollivirus*, virion walls are composed of closely applied layers that differ in electron density. In *Pandoravirus*, the middle of three layers consists of a “dense mesh of fibrils,” [4]. In *Mollivirus*, the inner of two layers has a similar meshwork construction and the outer layer is formed of parallel strips, spaced 30- to 40-nm apart and oriented tangential to the surface [6]. None of these features are evident in capsids of *Meelsvirus*—the capsids are not layered, no fibrillary mesh is evident, and there are no corresponding tangentially oriented parallel strips. The 60 nm-thick wall of *Pithovirus* virions consists of a solid-looking electron-dense matrix within which are embedded 10 nm-spaced radial columns of a less electron-dense material [5]. The wall of *Pithovirus* thickens by accretion of globules of these materials onto the outside of the thickening wall, rather than self-organization of subunits at a growing edge, as occurs in *Meelsvirus*. Based on ultrastructural and biochemical differences among *Pandoravirus* and *Pithovirus*, Legendre et al. [5], deemed their ovoid shape to be the result of convergence rather than common descent. The same is probably true for the ovoid shape of *Meelsvirus* capsids.

Several ultrastructural studies of non-giant virions provide clues for understanding the patterned substructure of *Meelsvirus* capsids. In *Vaccinia* virus (Poxviridae), the 10–15 nm thick capsids show a simple striated pattern when viewed by conventional TEM because each capsomer has an electron dense spicule projecting radially [37]. A similar situation exists in *Herpes simplex* virus-1 (Herpesviridae) [38, 39]. The substructure of the *Meelsvirus* capsid is much more elaborate than in either of the latter two species, but the radial electron-dense component of *Meelsvirus* capsomers may correspond to spicular elaborations of capsomers. Alternatively, thickness of the *Meelsvirus* capsid may be the result of protein stacking. In some viruses (Herpesviridae, for example), the forming procapsid appears thicker than the final capsid because assembling major capsid proteins are associated with "scaffold proteins" on their inner side. The latter separate from capsomers prior to filling with the viral genome [40]. From an evolutionary perspective, it is plausible that thickness of *Meelsvirus* capsids is achieved by having scaffold-like proteins become a permanent part of the finished capsid and, thus, part of a larger more complex type of capsomer.

### Stemmed conical tail

Tails, as unilateral or terminal extensions, exist in diverse viruses, but they vary in structure and likely evolved independently in different virus lineages [41–43]. The conical tail of *Meelsvirus* is an extension of the envelope and, thus, structurally distinct from the cylindrical tail of the giant virions of *Tupanvirus* (family Mimiviridae) [7]. The latter lack an external envelope and the tail is a separate structure adhering to the outside of the capsid. Likewise, the tail of *Meelsvirus* bears no structural resemblance to the tail of phages, which are non-membranous assemblages of proteins forming a narrow tube that connects to the capsid [41]. Among large nuclear viruses, *Whispovirus* is the only one we know of that has a tail formed by extension of an envelope [29, 43, 44]. It is the sole member of the family Nimaviridae and causes white spot syndrome in shrimp. *Whispovirus* is much smaller than *Meelsvirus* and has a very different capsid morphology [43]. Its tail, which has not been described in additional detail, is reported to function like a bacterial flagellum [45]. It seems unlikely that the tail of *Meelsvirus* has a motility function. Genetic analyses are needed to determine whether these viruses are related.

In *Meelsvirus*, elaboration of the envelope as a tail creates an enlarged compartment for accumulation of extracapsidular molecules. From a morphological perspective, this compartment potentially exists in all enveloped viruses. For example, the so-called lateral bodies of *Vaccinia* (Poxviridae) are situated between the envelope and nucleocapsid [37]. They contain viral proteins (enzymes) that are delivered to the host cytosol during infection [46]. Likewise, the tegument proteins of *Herpes simplex* viruses are positioned between the nucleocapsid and the envelope membrane [38] and are delivered to the host cytosol during infection [47]. The same may be true for the contents of the tail of *Meelsvirus*.

### Self-assembly of envelope coincident with self-assembly of capsid

*Meelsvirus* is unusual among nuclear viruses in having the envelope “self-assemble” coincidently with the capsid. For enveloped nuclear viruses, fully formed nucleocapsids commonly become surrounded by host cell membrane as they bud through the nuclear envelope on the way to the cytoplasm or as they exit the host cell [48–50]. So far, nuclear egress has not been observed for *Meelsvirus* (the few intact cytoplasmic viruses may have reached the cytoplasm through a rupture in the nuclear envelope). In *Meelsvirus*, morphogenesis of the envelope appears to be spatially separated from all host cell membranes, and thus, appears to be formed *de novo*. Whether or not lipid membranes can arise *de novo* is still an open question of biophysical interest because it is central to the question of the early evolution of cellular membranes [51]. Apparent *de novo* synthesis of membranes associated with cytoplasmic viruses have been debunked [52, 53], but the question remains open for certain nuclear viruses (e.g., Baculoviridae) [54].

Several lines of evidence suggest that the *Meelsvirus* envelope contains a unit membrane. First, the central dense layer of the *Meelsvirus* envelope is very similar in thickness to the unit membranes of the host cell’s nuclear envelope. Second, the wavy form of the envelope where it surrounds the nucleocapsid suggests that it, like unit membranes, is flexible. Third, the envelope of *Meelsvirus* may have a transport function during tail formation and may be semipermeable. Because the extracapsidular space is "empty" during formation of the capsid and envelope, we infer that the electron dense contents of the tail are accumulated only after the completely enveloped capsid separates from the peripheral electron dense nucleoplasm. We can think of two possible origins for the extracapsidular material in the tail of *Meelsvirus*. It may be extruded from the nucleocapsid through the portal-like specialization that borders the tail or it may be accumulated from the surrounding nucleoplasm by active transport through the envelope. If the latter is true, the ability of the envelope of finished virus particles to retain the contents of the tail would provide evidence that the envelope is semipermeable.

Assuming that the central membrane of the envelope is a lipid bilayer, the source of the lipid molecules remains an open question. It could be assembled from host membrane lipid molecules, possibly those released by disruption/dissolution of the apical part of the nuclear envelope. The chemical composition and method of self-assembly of the envelope of *Meelsvirus* deserves further study.

### Infectious cycle

We can envision two different mechanisms for transmission of *Meelsvirus* to new hosts. Most simply, protruding virion-filled nuclei may rupture, causing the virions to be freely dispersed in sea water. Infection of new hosts would presumably involve chance encounters between swimming arrow worms and infective virions. Assuming a low concentration of virions in the environment, the probability of encounters between virions and arrow worms would be extremely small.

Alternatively, *Meelsvirus* may have an unconventional mode of transmission that depends on the mating biology of arrow worms. *Adhesisagitta hispida*, like other chaetognaths, is hermaphroditic and sperm are transferred during a fairly elaborate "mating dance" [55]. A male-acting individual slaps the posterior end of its body, including the sperm-filled seminal receptacles, against the mate’s body. For well-fed individuals, mating can happen as often as daily [56]. We postulate that protruding virus-infected nuclei rupture upon physical contact with the mate’s epidermis, spreading a cluster of infective viral particles over a small areas of the mate’s body. This possible mode of viral infection is consistent with the following observations. Both of our infected animals are mature and had recently mated, as evidenced by the presence of sperm in the ovaries. For each host specimen, the infected epidermal cells are at a similar stage of “attack” by *Meelsvirus*, suggesting that they became infected simultaneously. Also, the infected cells are grouped in a small area of the host’s epidermis. Such a mode of viral transfer would ensure host specificity and result in relatively high rates of infection compared to the simpler dispersal mode.

The mechanism by which *Meelsvirus* enters a host cell almost certainly differs from that of other known giant viruses. In the latter, virions are phagocytosed and, consequently, become enclosed within a phagosome [4–7, 12]. The genomic contents of capsids are released to the host cytoplasm when the phagosome membrane fuses with a viral membrane located just inside the capsid wall; this occurs through a portal that opens in the capsid wall [4–7, 57]. For *Meelsvirus*, the naked capsids in the cytoplasm of infected host cells are probably the remains of the originally infecting virions, but they are not enclosed by membrane, revealing that they were not phagocytosed. Furthermore, virions of *Meelsvirus* lack an internal membrane, which is a key component of the infection mechanism of other giant viruses [12]. Typically, in viruses having an external envelope, the envelope participates in infection by fusing with the host cell membrane, thereby delivering a naked capsid directly into the host cell’s cytoplasm [50]. The absence of an envelope around the naked cytoplasmic capsids of *Meelsvirus* is consistent with the hypothesis that it plays a similar role during infection. Possibly, the envelope fuses with the host plasma membrane, thereby delivering both the capsid and extracapsidular contents of the tail into the host cell.

## Future work

Fresh specimens of *Meelsvirus* are needed to address the many questions raised by our ultrastructural study. Obvious problems needing resolution include the molecular structure of the capsid wall, whether or not the envelope is a lipid bilayer equivalent to the unit membranes of cells, the mechanism of infection of new hosts, function of the tail and its contents, genomic composition of the virus, and evolutionary relationship of *Meelsvirus* to other viruses.

Although we have not attempted to do so, we expect that *Meelsvirus* can be propagated in an appropriate marine laboratory setting (e.g., coastal Florida). The host, *Adhesisaggita hispida*, is a near shore species that is readily collected from appropriately located fishing piers and can be kept alive in the lab for many days [55, 58]. Although we discovered *Meelsvirus* using transmission electron microscopy, infected epidermal cells can probably be detected by careful inspection of live arrow worms using a compound microscope. The 1 cm-long adults of *A*. *hispida* are small enough to fit beneath a cover glass on a microscope slide, and their transparency makes anatomical details easy to see [59]. For our thick sections, using a 40X objective, virus-infected nuclei are clearly visible and the individual virions are just barely visible. The replication cycles of other giant viruses are fairly short: 6–24 hours from infection to release of new virions [4–6, 35]. If the same is true for *Meelsvirus*, survival of hosts in the laboratory should not be a limiting factor. If formed virions are shed into the water, either individually or enclosed in nuclei, they should be visible in the culture vessels. If virions are transmitted during mating, this could be observed directly or, perhaps, uninfected individuals could be experimentally infected by physical manipulation of hosts.
